# Construction and topological analysis of an endometriosis-related exosomal circRNA-miRNA-mRNA regulatory network

**DOI:** 10.18632/aging.202937

**Published:** 2021-04-26

**Authors:** Jingni Wu, Xiaoling Fang, Hongyan Huang, Wei Huang, Lei Wang, Xiaomeng Xia

**Affiliations:** 1 Department of Obstetrics and Gynecology, The Second Xiangya Hospital, Central South University, Changsha 410011, Hunan, China; 2 Research Center of Carcinogenesis and Targeted Therapy, Xiangya Hospital, Central South University, Changsha 410008, Hunan, China; 3 The Higher Educational Key Laboratory for Cancer Proteomics and Translational Medicine of Hunan Province, Xiangya Hospital, Central South University, Changsha 410008, Hunan, China; 4 NHC Key Laboratory of Carcinogenesis and the Key Laboratory of Carcinogenesis and Cancer Invasion of the Chinese Ministry of Education, Cancer Research Institute, School of Basic Medical Science, Central South University, Changsha 410078, Hunan, China

**Keywords:** endometriosis, exosome, biomarker, circRNA, topological analysis

## Abstract

Novel biomarkers are needed to accelerate the diagnosis and treatment of endometriosis. We performed RNA sequencing to explore the expression profiles of exosomal circular RNAs (circRNAs), microRNAs (miRNAs) and mRNAs in patients with ovarian endometriomas, eutopic endometria and normal endometria. Differentially expressed genes between the different pairs of groups were analyzed and functionally annotated. Then, miRNA-target RNA pairs were identified, competing endogenous RNA (ceRNA) scores were calculated, gene expression characteristics were determined, and these parameters were used to construct an exosomal ceRNA network. We identified 36 candidate hub genes with high degrees of gene connectivity. We also topologically analyzed the ceRNA network to obtain a hub ceRNA network of circRNAs with the highest closeness and ceRNA efficiency. Twelve genes overlapped between the 36 candidate hub genes and the genes in the hub ceRNA network. These 12 genes were considered to be exosomal RNA-based biomarkers, and circ_0026129/miRNA-15a-5p/ATPase H+ transporting V1 subunit A (*ATP6V1A*) were at the center of the ceRNA network. By determining the exosomal RNA expression profiles of endometriosis patients and constructing a circRNA-associated ceRNA network, these findings provide insight into the molecular pathways of endometriosis and new resources for its diagnosis and treatment.

## INTRODUCTION

Endometriosis is a common, non-malignant gynecological disorder characterized by the growth of endometrial glands and stroma outside the uterus [1]. Up to 80% of women with chronic pelvic pain may have endometriosis, along with around 50% of women being treated for infertility [2, 3]. However, the pathogenesis of endometriosis is not well characterized, and there are often delays in diagnosing patients, leading to difficulties in medical and surgical treatments [4]. Therefore, it is essential to discover novel diagnostic biomarkers and therapeutic methods for endometriosis.

Noncoding RNAs such as circular RNAs (circRNAs) and microRNAs (miRNAs) are functional RNA molecules without protein-coding capacity. MiRNAs can induce the degradation and inhibit the translation of their target mRNAs [5]. CircRNAs are covalently closed-loop structures generated from mRNAs through a splicing-like process (back-splicing). According to the competing endogenous RNA (ceRNA) theory, circRNAs can competitively bind to miRNAs as endogenous molecular sponges, ultimately altering mRNA expression. This circRNA regulatory mechanism indicates that there are communication networks among RNAs for regulating each other’s expression via competing shared target miRNAs.

CeRNA networks involving circRNAs, miRNAs and mRNAs have been detected in many diseases, including cancer and Alzheimer’s disease [6–8]. Systematic analysis of ceRNA network could significantly improve the functional understanding of coding and noncoding RNAs. In addition, changes in topologically important ceRNAs have been associated with the pathogenesis and treatment of lung adenocarcinoma, osteoarthritis, clear cell renal cell carcinoma and so on [9–11]. Hsa_circ_0067301 was identified as a ceRNA that suppresses Notch-1 expression by increasing miR-141 expression, and the downregulation of this circRNA was found to promote the epithelial-mesenchymal transition in endometriosis [12]. Topologically important hsa_circ_0002286/has-mir-222-5p/TRIM2 axis played a critical role in the progression of clear cell renal cell carcinoma [11]. However, there have been few studies of ceRNA networks in endometriosis.

Exosomes are a subset of extracellular vesicles that are critical for cellular communication [13]. They range in size from 30 to 150 nm, and transfer RNAs, lipids and proteins to recipient cells, thus participating in multiple diseases [14–16]. Exosomal RNAs are considered to be more functional than other RNAs because they are protected from RNase [17]. Therefore, exosomes may be a suitable setting for RNA crosstalk, and provide an ideal study model for the ceRNA hypothesis. Exosomal RNA biomarkers have been identified in various tumors [18, 19], and exosomes have been detected in all biological fluids [20], suggesting that exosomal RNAs could be used to diagnose and treat diseases [21, 22]. Exosomes secreted from endometriotic stromal cells can transfer information to other cells, contributing to the pathogenesis of endometriosis [23, 24]. However, little is known about the functions of exosomes or exosomal circRNA-miRNA-mRNA regulatory networks in endometriosis.

In this study, we derived exosomes from endometrial stromal cells, and used RNA sequencing to systematically analyze exosomal circRNA, miRNA and mRNA levels. Then, we constructed an exosomal ceRNA network, and topologically analyzed it to screen out hub RNAs for the construction of a hub ceRNA network. Finally, we identified the central genes in the ceRNA network, and used quantitative real-time PCR (qRT-PCR) to validate their expression in endometriosis. Our findings have clarified the molecular mechanisms of endometriosis and provided valuable information for its diagnosis and treatment.

## RESULTS

### Differentially expressed exosomal circRNAs, miRNAs and mRNAs in endometriosis

**Figure 1:**
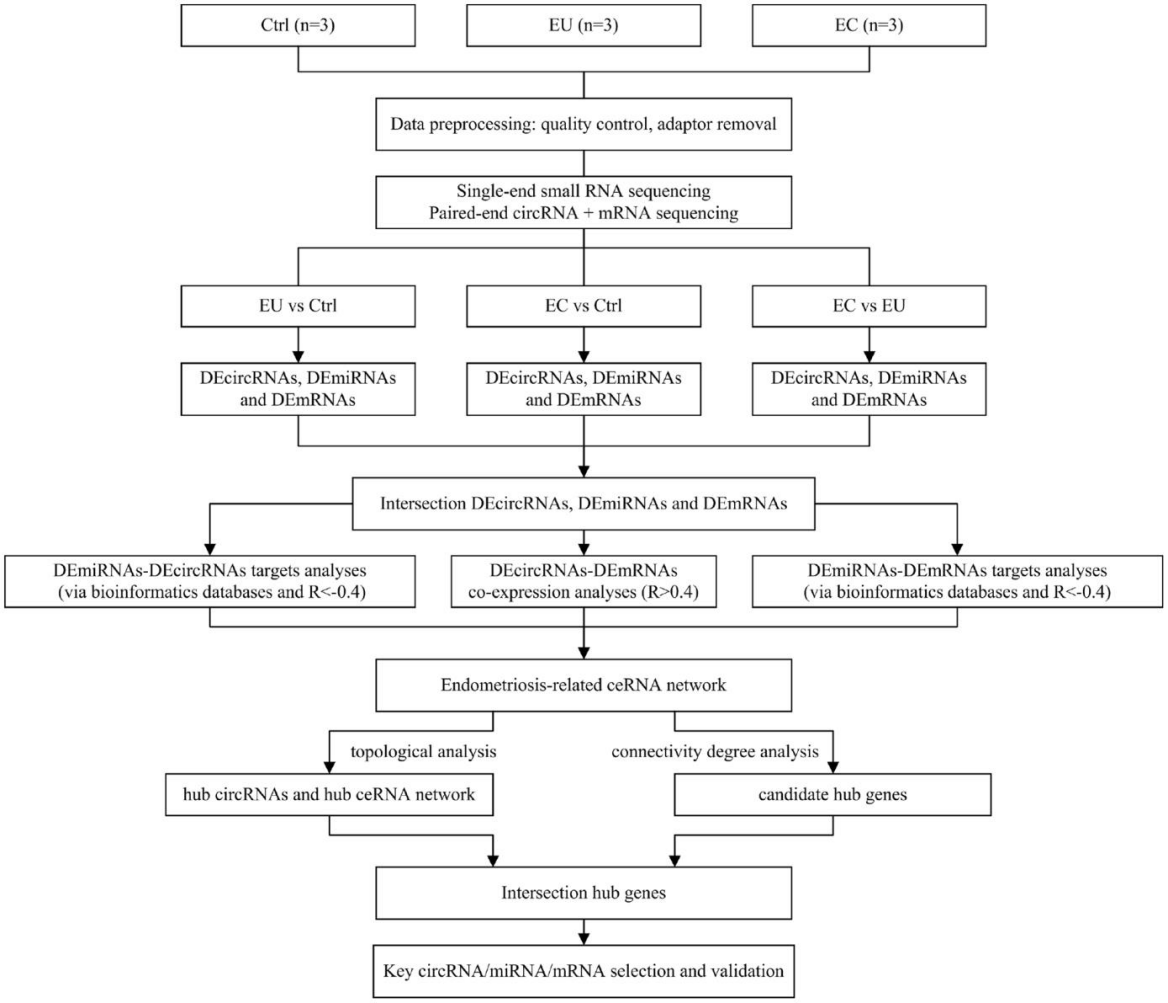
Flowchart of the data preparation, preprocessing and analysis strategy.

**Figure 2:**
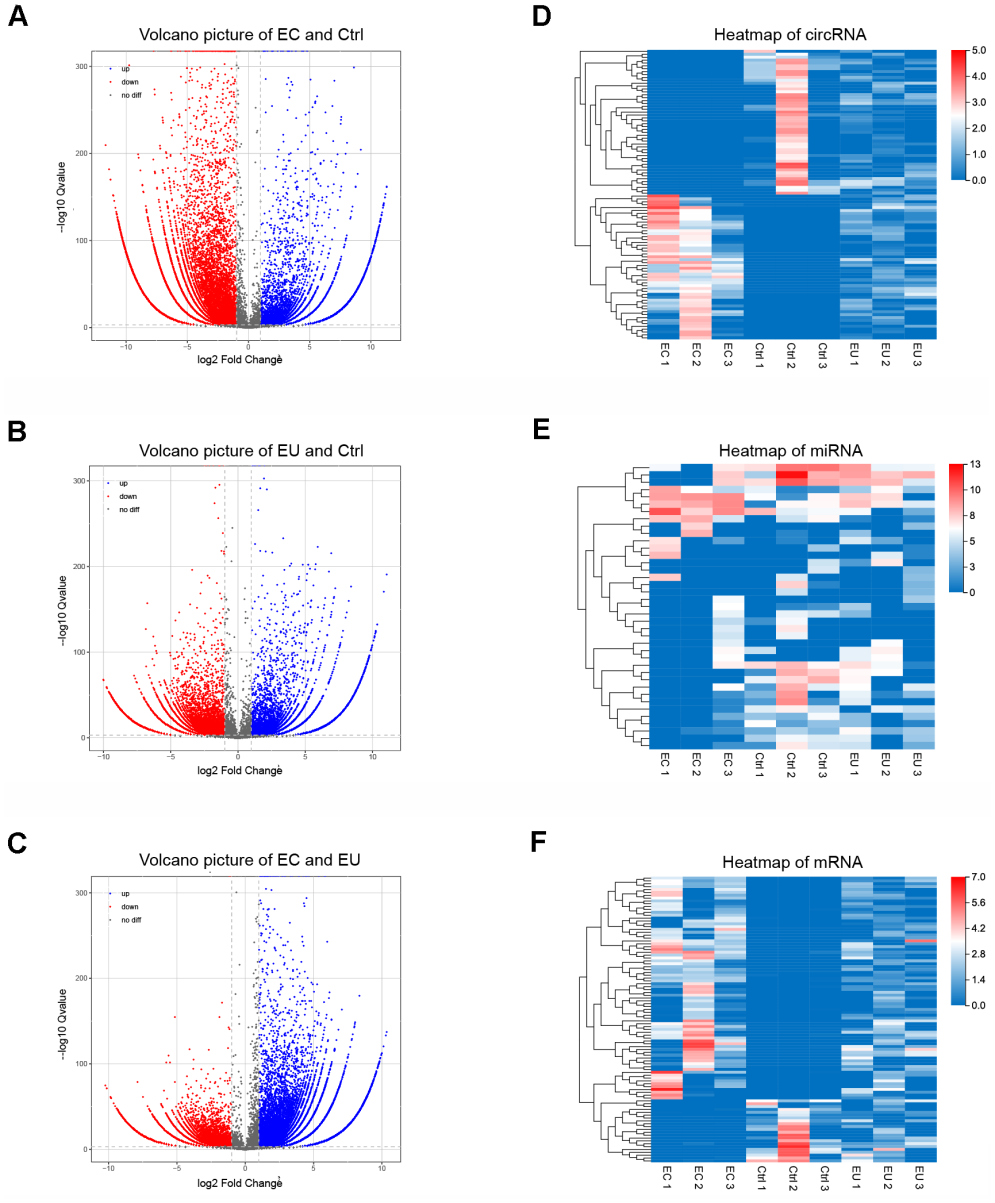
**Identification of exosomal DECs, DEMis and DEMs in endometriosis.** (**A**–**C**) Volcano plots of exosomal DECs based on the |log fold-change| between the EC and Ctrl groups (**A**), the EU and Ctrl groups (**B**) and the EC and EU groups (**C**). (**D**–**F**) Heatmap showing the expression of overlapping DEGs in the EC vs. Ctrl, EU vs. Ctrl and EC vs. EU comparisons, including the top 100 DECs, top 100 DEMis and top 100 DEMs. DECs, DEMis and DEMs: Differentially expressed circRNAs, miRNAs and mRNAs.

The flowchart of our work is shown in Figure 1. To identify differentially expressed exosomal circRNAs, miRNAs and mRNAs in endometriosis, we performed RNA sequencing on endometrial stromal cell-secreted exosomes from patients with ovarian endometriomas (EC), eutopic endometria (EU) and normal endometria (Ctrl). The top 10 differentially expressed circRNAs (DECs), miRNAs (DEMis) and mRNAs (DEMs) between the EC and Ctrl groups, between the EU and Ctrl groups, and between the EC and EU groups are shown as heatmaps (Supplementary Figure 1A–1C). The differences in circRNA expression between the respective pairs of groups are shown in Volcano plots (Figure 2A–2C).

Overlapping differentially expressed genes (DEGs) among the three comparison sets (EC vs. Ctrl, EU vs. Ctrl and EC vs. EU) were identified based on a log2 (fold-change) ≥ 1 and a corrected P-value < 0.001. Using these cutoff criteria, we identified 6512 overlapping circRNAs (2915 upregulated and 640 downregulated in all three comparison sets), 39 overlapping miRNAs (17 upregulated and 9 downregulated in all three comparison sets) and 1449 overlapping mRNAs (550 upregulated and 136 downregulated in all three comparison sets). These overlapping DEGs were used for subsequent analyses. Heatmaps of the overlapping DEGs revealed distinct RNA expression patterns in exosomes from EC, EU and Ctrl patients (Figure 2D–2F). Our results indicated that RNAs enriched in endometrial stromal cell-secreted exosomes may be involved in the etiology of endometriosis.

### Functional enrichment analysis of DEMs

**Figure 3:**
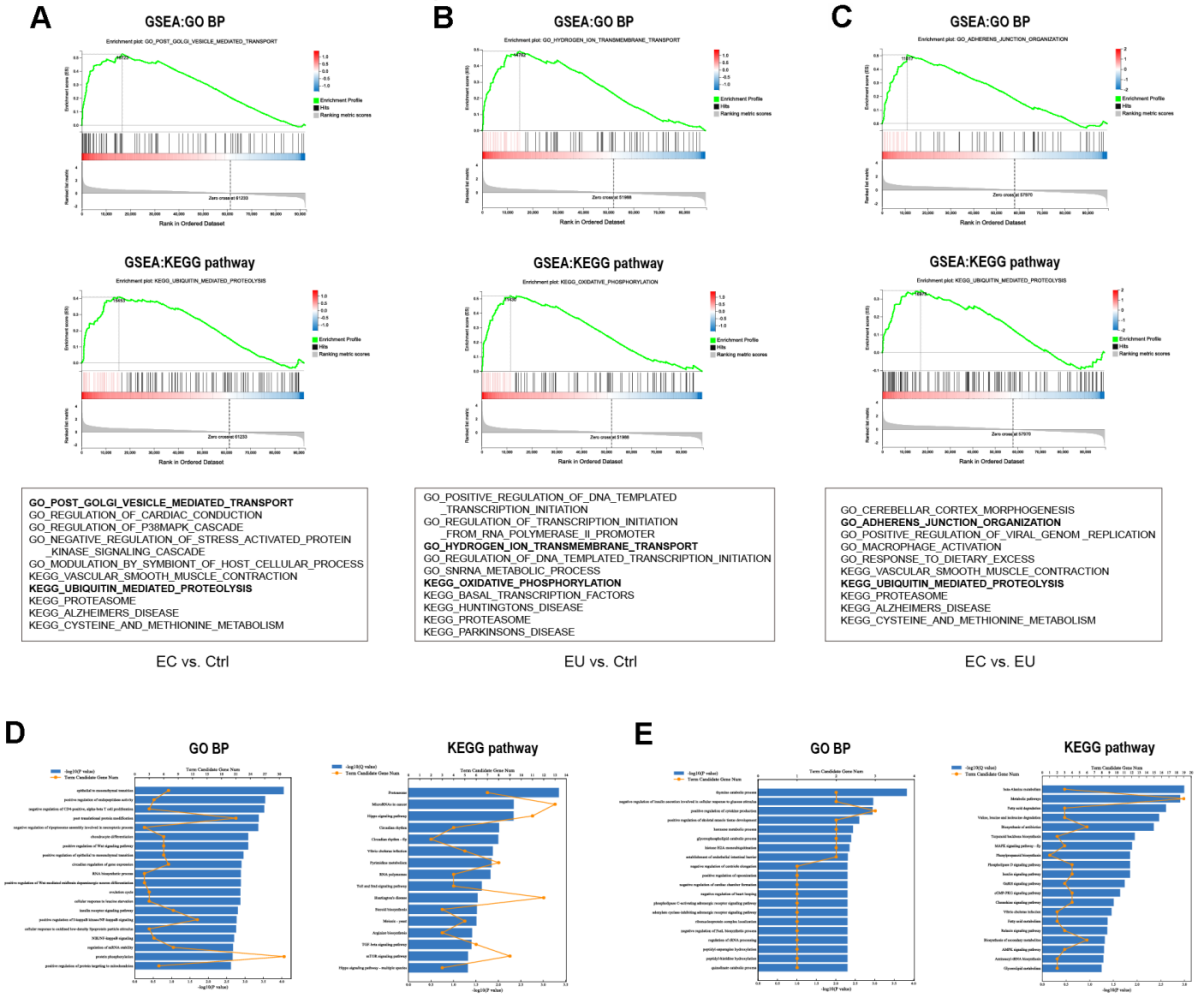
**Functional enrichment analysis of DEMs.** (**A**–**C**) The top 5 C5 GO biological processes and top 5 C2 KEGG pathways enriched in the DEMs between the EC and Ctrl groups (**A**), the EU and Ctrl groups (**B**) and the EC and EU groups (**C**) in the GSEA. The six most common functional gene sets in endometriosis are shown. (**D**, **E**) GO and KEGG pathway analyses of the overlapping upregulated DEMs (**D**) and downregulated DEMs (**E**) among the three comparison sets. GO: Gene ontology. BP: Biological process. KEGG: Kyoto Encyclopedia of Genes and Genomes.

Next, we performed a gene set enrichment analysis (GSEA) to annotate the functions of the DEMs from the above pairwise comparisons. The top five enriched Kyoto Encyclopedia of Genes and Genomes (KEGG) pathways and the top five enriched Gene Ontology (GO) biological processes from the three comparisons are shown in Figure 3A–3C and Supplementary Table 1. We also performed GO and KEGG pathway analyses on the overlapping DEMs from the three comparison sets (Figure 3D, 3E). The upregulated DEMs were mainly enriched in the ‘epithelial to mesenchymal transition’, ‘positive regulation of endopeptidase activity’, ‘proteasome pathway’ and ‘Hippo signaling pathway’ (Supplementary Table 2), while the downregulated DEMs were enriched in the ‘negative regulation of insulin secretion involved in cellular response to glucose stimulus’, ‘positive regulation of cytokine production’, ‘metabolic pathway’ and ‘fatty acid degradation pathway’ (Supplementary Table 3). These functions are known to be associated with endometriosis [25, 26], supporting our RNA sequencing analysis.

### Prediction of miRNA-target interactions and ceRNA pairs

**Figure 4:**
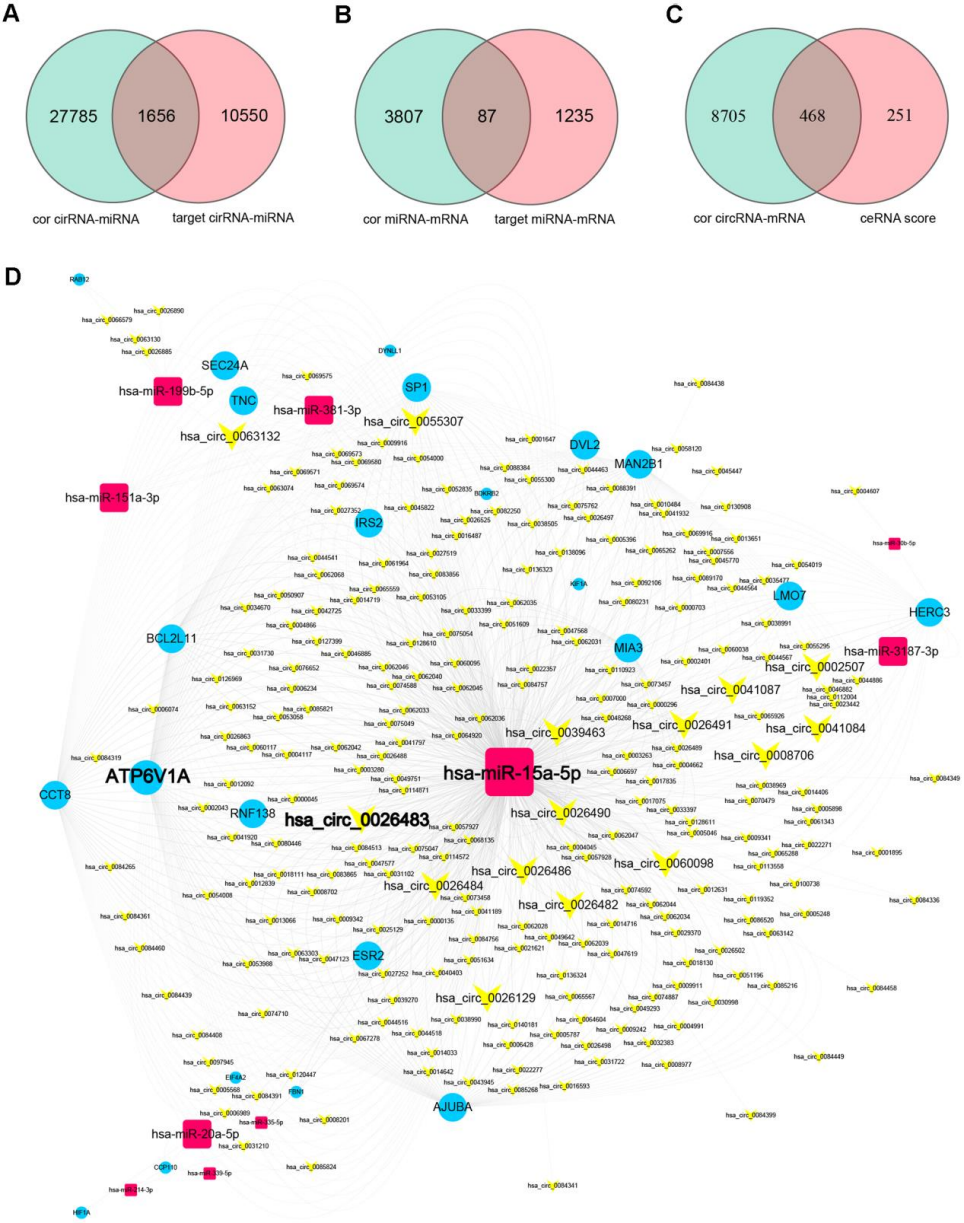
**Prediction of miRNA-target interactions and ceRNA pairs, and construction of a circRNA-miRNA-mRNA ceRNA regulatory network.** (**A**) MiRNA-circRNA interactions were filtered based on the miRNA-circRNA correlation coefficients and the miRNA-circRNA target pairs. (**B**) MiRNA-mRNA interactions were filtered based on the miRNA-mRNA correlation coefficients and the miRNA-mRNA target pairs. (**C**) Filtered miRNA-target interactions were integrated into circRNA-mRNA pairs (ceRNA pairs) based on shared miRNAs. CeRNA pairs were further filtered based on the ceRNA scores and the mRNA-circRNA correlation coefficients. (**D**) The ceRNA network. Yellow V-shaped nodes represent DECs, red rectangular nodes represent DEMis, and blue circular nodes represent DEMs. Larger nodes in the ceRNA network represent genes with higher degrees of connectivity.

Subsequently, using the DEGs that overlapped among the three comparison sets (EC vs. Ctrl, EU vs. Ctrl and EC vs. EU), we determined the Pearson correlation coefficients between miRNAs and circRNAs/mRNAs, and used bioinformatic databases to predict miRNA-circRNA and miRNA-mRNA pairs. We then selected the overlapping miRNA-target pairs between the correlation analysis and the bioinformatic analysis. We obtained 1656 overlapping miRNA-circRNA pairs (including 25 miRNAs and 1154 circRNAs) from 29,441 miRNA-circRNA interactions (R < -0.4) and 12,206 miRNA-circRNA filtered target pairs (Figure 4A). In addition, we obtained 87 overlapping miRNA-mRNA pairs (including 25 miRNAs and 60 mRNAs) from 3894 miRNA-mRNA interactions (R < -0.4) and 1322 miRNA-mRNA filtered target pairs (Figure 4B).

Thereafter, we integrated these pairs based on shared miRNAs, and thus obtained 345,118 candidate circRNA-miRNA-mRNA competing interactions from the overlapping DEGs among the three comparison sets. When we filtered these competing interactions based on the circRNA-mRNA correlation (R > 0.4) and the ceRNA score (score > 0.3, P < 0.05), we identified 468 circRNA-mRNA ceRNA pairs (including 247 circRNAs and 23 mRNAs) (Figure 4C) and 10 shared miRNAs. The top 10 ceRNA pairs based on the ceRNA scores are shown in Table 1.

**Table 1:** The top 10 ceRNA pairs based on the ceRNA score.

**circRNA**	**mRNA**	**circ_m r**	**ceRNA Score**	**ceRNA P-value**
hsa_circ_0026129	*ATP6V1A*	0.93917092	1	0.001059883
hsa_circ_0140181	*ATP6V1A*	0.823446797	1	0.001059883
hsa_circ_0026129	*MAN2B1*	0.802468977	1	0.001059883
hsa_circ_0014716	*DVL2*	0.831070515	1	0.001287001
hsa_circ_0110923	*DVL2*	0.695248588	1	0.001287001
hsa_circ_0080231	*HERC3*	0.516167256	1	0.00190779
hsa_circ_0048268	*CCT8*	0.936345086	1	0.002574003
hsa_circ_0000296	*CCT8*	0.913827698	1	0.002574003
hsa_circ_0026129	*RNF138*	0.620632147	1	0.00317965
hsa_circ_0014716	*LMO7*	0.729299492	1	0.004504505

ceRNA pair: circRNA-mRNA pair. circ_m r: the correlation coefficient between the circRNA and mRNA levels.

### Construction of co-expression and ceRNA networks

We then constructed a co-expression network using the 468 ceRNA pairs identified above (Supplementary Figure 2), and established a ceRNA regulatory network using the 468 ceRNA pairs and 10 shared miRNAs (Figure 4D). The ceRNA network contained 247 circRNA nodes, 23 mRNA nodes, 10 miRNA nodes and 936 edges. It is well known that hub nodes with high degrees of connectivity have vital functions in biological networks [27, 28]. Based on these studies and previous reports on genes associated with endometriosis, we identified 36 candidate hub nodes (degree ≥ 5) in the ceRNA network, including 15 circRNAs, 6 miRNAs and 15 mRNAs (Table 2 and Figure 4D).

**Table 2:** Differentially expressed genes with high degrees of connectivity (node degrees ≥ 5).

**Number**	**Gene type**	**Gene name**	**Genbank description**	**Node degrees**
1	miRNA	hsa-miR-15a-5p	NA	876
2	mRNA	*ATP6V1A*	ATPase H+ transporting V1 subunit A	87
3	mRNA	*CCT8*	Chaperonin containing TCP1 subunit 8	72
4	mRNA	*BCL2L11*	BCL2-like 11	66
5	mRNA	*AJUBA*	Ajuba LIM protein	45
6	mRNA	*MAN2B1*	Mannosidase alpha class 2B member 1	42
7	mRNA	*HERC3*	HECT and RLD domain containing E3 ubiquitin protein ligase 3	32
8	mRNA	*SP1*	Sp1 transcription factor	28
9	mRNA	*MIA3*	MIA SH3 domain ER export factor 3	21
10	mRNA	*DVL2*	Disheveled segment polarity protein 2	19
11	miRNA	hsa-miR-381-3p	NA	18
12	miRNA	hsa-miR-3187-3p	NA	12
13	mRNA	*RNF138*	Ring finger protein 138	10
14	circRNA	hsa_circ_0026129	NA	8
15	mRNA	*LMO7*	LIM domain 7	8
16	miRNA	hsa-miR-199b-5p	NA	8
17	circRNA	hsa_circ_0039463	NA	7
18	miRNA	hsa-miR-20a-5p	NA	6
19	circRNA	hsa_circ_0055307	NA	6
20	circRNA	hsa_circ_0041084	NA	6
21	circRNA	hsa_circ_0041087	NA	6
22	circRNA	hsa_circ_0002507	NA	6
23	circRNA	hsa_circ_0026483	NA	6
24	circRNA	hsa_circ_0008706	NA	6
25	circRNA	hsa_circ_0026490	NA	6
26	miRNA	hsa-miR-151a-3p	NA	6
27	mRNA	*SEC24A*	SEC24 homolog A, COPII coat complex component	6
28	circRNA	hsa_circ_0063132	NA	5
29	mRNA	*ESR2*	Estrogen receptor 2	5
30	circRNA	hsa_circ_0026486	NA	5
31	circRNA	hsa_circ_0060098	NA	5
32	mRNA	*IRS2*	Insulin receptor substrate 2	5
33	circRNA	hsa_circ_0026491	NA	5
34	circRNA	hsa_circ_0026484	NA	5
35	circRNA	hsa_circ_0026482	NA	5
36	mRNA	*TNC*	Tenascin C	5

### Topological analysis and construction of a hub ceRNA sub-network

**Figure 5:**
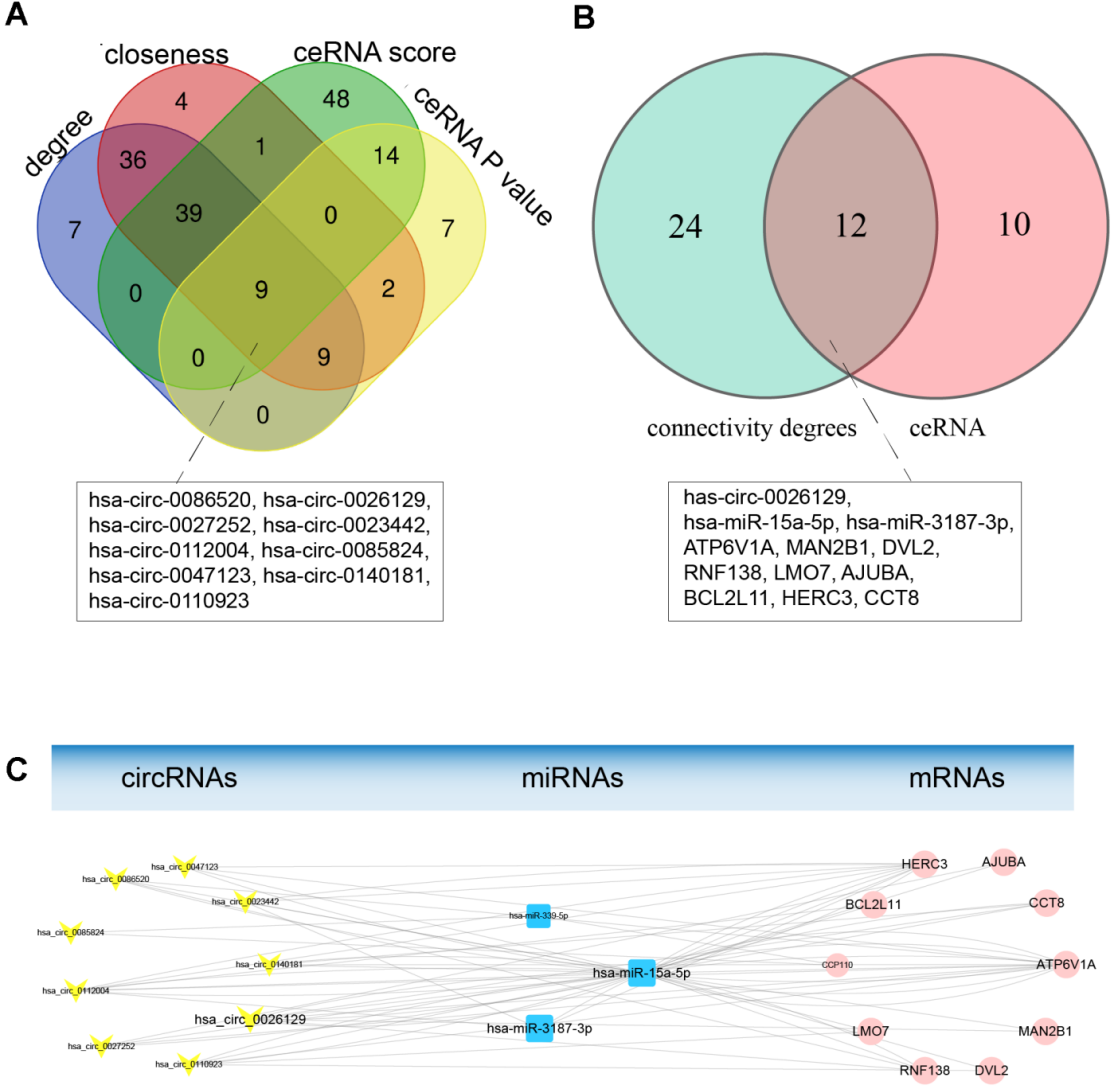
**Topological analysis and construction of the hub ceRNA sub-network.** (**A**) Topological characteristics of the ceRNA netwok. Nine hub circRNAs overlapped among the lists of genes with ceRNA scores > 0.7, genes with ceRNA P-values < 0.01 and the top 100 genes based on the degree of connectivity and betweenness. (**B**) Venn diagram of overlapping genes between the hub ceRNA network of nine circRNAs and the genes with higher degrees of connectivity. (**C**) The hub ceRNA sub-network containing the nine hub circRNAs. Yellow V-shaped nodes represent DECs, red rectangular nodes represent DEMis, and blue circular nodes represent DEMs. The larger nodes represent the overlapping hub genes.

To construct a hub ceRNA sub-network, we analyzed the topological characteristics of the ceRNA network based on degree, closeness, ceRNA score and ceRNA P-value. Nine circRNAs overlapped among the top-ranking lists for these four characteristics, as shown in Figure 5A. We extracted the first miRNA neighbors and second mRNA neighbors of these hub circRNAs from the ceRNA network. Interestingly, these extracted mRNAs and miRNAs also had high degrees of connectivity and closeness. Hence, we used these genes to construct a hub ceRNA sub-network, which included 9 circRNA nodes, 3 miRNA nodes, 10 mRNA nodes and 66 edges (Figure 5C and Table 3).

**Table 3:** The topological characteristics of the hub ceRNA network.

**circRNA**	**miRNA**	**mRNA**	**ceRNA score**	**ceRNA P-value**	**circRNA closeness**	**circRNA degree**	**miRNA closeness**	**miRNA degree**	**mRNA closeness**	**mRNA degree**
hsa_circRNA_0023442	hsa-miR-15a-5p	*HERC3*	0.71429	0.00486	0.001739	2	0.003236	876	0.001751	32
hsa_circRNA_0023442	hsa-miR-3187-3p	*HERC3*	0.71429	0.00486	0.001739	2	0.001209	12	0.001751	32
**hsa_circRNA_0026129**	**hsa-miR-15a-5p**	***MAN2B1***	**1**	**0.00106**	**0.001733**	**8**	**0.003236**	**876**	**0.001733**	**42**
**hsa_circRNA_0026129**	**hsa-miR-15a-5p**	***ATP6V1A***	**1**	**0.00106**	**0.001733**	**8**	**0.003236**	**876**	**0.001733**	**87**
**hsa_circRNA_0026129**	**hsa-miR-15a-5p**	***RNF138***	**1**	**0.00318**	**0.001733**	**8**	**0.003236**	**876**	**0.001733**	**10**
**hsa_circRNA_0026129**	**hsa-miR-15a-5p**	***CCT8***	**0.75**	**0.00961**	**0.001733**	**8**	**0.003236**	**876**	**0.001733**	**72**
hsa_circRNA_0027252	hsa-miR-15a-5p	*AJUBA*	1	0.00721	0.001733	4	0.003236	876	0.001733	45
hsa_circRNA_0027252	hsa-miR-15a-5p	*ATP6V1A*	1	0.00721	0.001733	4	0.003236	876	0.001733	87
hsa_circRNA_0047123	hsa-miR-15a-5p	*ATP6V1A*	0.71429	0.00229	0.001733	3	0.003236	876	0.001733	87
hsa_circRNA_0047123	hsa-miR-15a-5p	*HERC3*	0.71429	0.00486	0.001733	3	0.003236	876	0.001751	32
hsa_circRNA_0085824	hsa-miR-339-5p	*CCP110*	0.75	0.00492	0.034483	1	0.047619	2	0.066667	4
hsa_circRNA_0086520	hsa-miR-15a-5p	*ATP6V1A*	0.71429	0.00229	0.001733	4	0.003236	876	0.001733	87
hsa_circRNA_0086520	hsa-miR-15a-5p	*RNF138*	0.71429	0.00915	0.001733	4	0.003236	876	0.001733	10
hsa_circRNA_0110923	hsa-miR-15a-5p	*DVL2*	1	0.00129	0.001733	3	0.003236	876	0.001733	19
hsa_circRNA_0110923	hsa-miR-15a-5p	*LMO7*	1	0.0045	0.001733	3	0.003236	876	0.001733	8
hsa_circRNA_0110923	hsa-miR-15a-5p	*ATP6V1A*	1	0.00721	0.001733	3	0.003236	876	0.001733	87
hsa_circRNA_0112004	hsa-miR-15a-5p	*BCL2L11*	0.8	0.00246	0.001739	4	0.003236	876	0.001733	66
hsa_circRNA_0112004	hsa-miR-15a-5p	*ATP6V1A*	0.8	0.00479	0.001739	4	0.003236	876	0.001733	87
hsa_circRNA_0112004	hsa-miR-15a-5p	*HERC3*	0.8	0.00838	0.001739	4	0.003236	876	0.001751	32
hsa_circRNA_0112004	hsa-miR-3187-3p	*HERC3*	0.8	0.00838	0.001739	4	0.001209	12	0.001751	32
hsa_circRNA_0140181	hsa-miR-15a-5p	*ATP6V1A*	1	0.00106	0.001733	3	0.003236	876	0.001733	87
hsa_circRNA_0140181	hsa-miR-15a-5p	*CCT8*	0.75	0.00961	0.001733	3	0.003236	876	0.001733	72

Next, we determined which genes overlapped between the hub ceRNA sub-network and the list of 36 candidate hub genes with higher degrees of connectivity, assuming that these were the most important genes in the ceRNA network. We identified 12 overlapping hub genes, including 1 circRNA, 2 miRNAs and 9 mRNAs, which we selected as endometriosis-associated exosomal biomarkers (Figure 5B and Table 3). MiRNA-15a-5p and ATPase H+ transporting V1 subunit A (*ATP6V1A*) have previously been associated with endometriosis [29, 30], and exhibited high topological significance in our analysis. Circ_0026129 was the top-ranking target of miRNA-15a-5p, and exhibited high ceRNA efficiency. Hence, circ_0026129, miRNA-15a-5p and *ATP6V1A* were considered to be the center of the ceRNA network.

### RT-PCR and miRNA target validation

**Figure 6:**
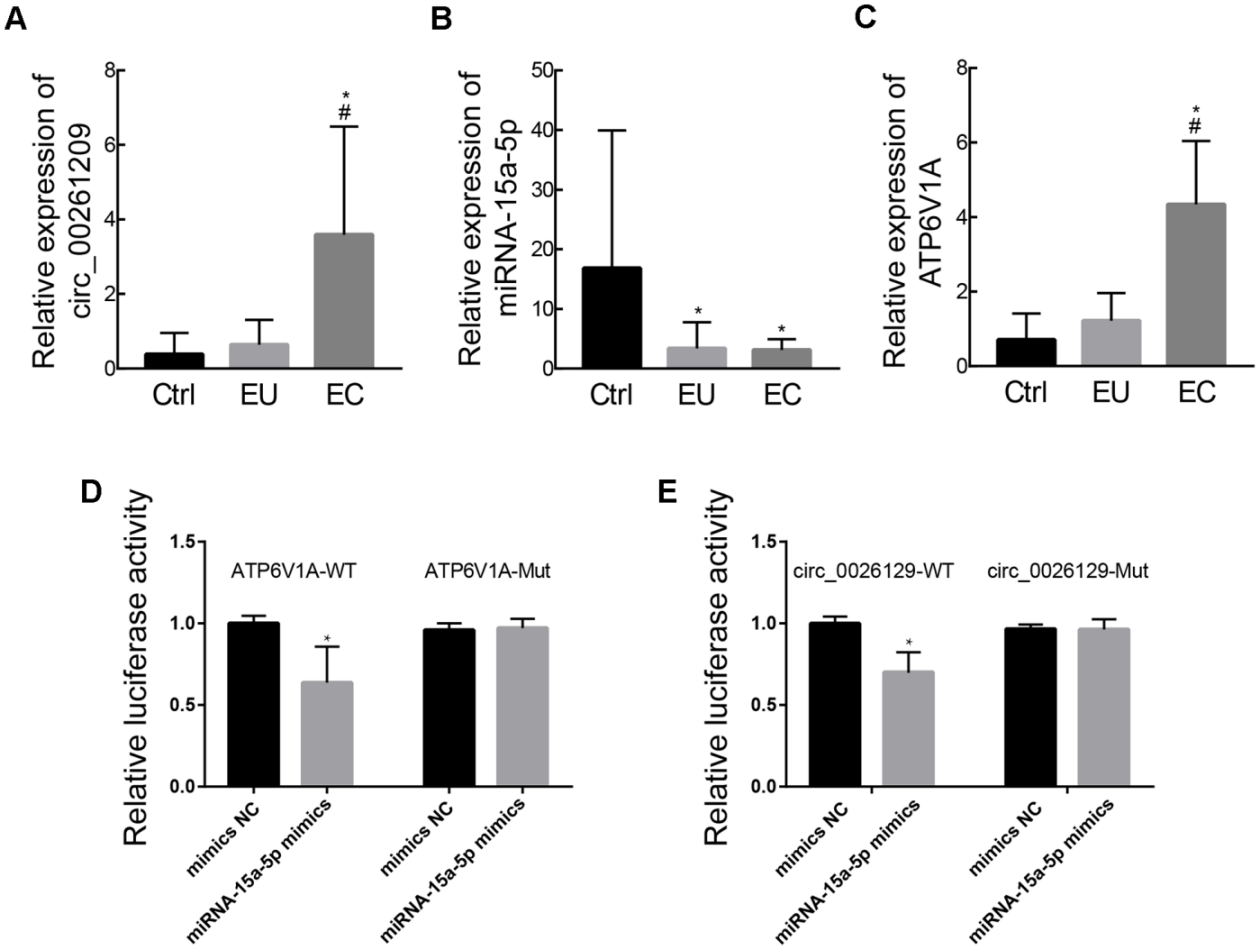
**Validation of exosomal circ_0026129, miRNA-15a-5p and *ATP6V1A* expression and their molecular binding in endometriosis.** (**A**–**C**) The relative expression of circ_0026129, miRNA-15a-5p and *ATP6V1A*, assessed using RT-PCR. (**D**, **E**) Luciferase reporter assay confirming that miRNA-15a-5p could bind to *ATP6V1A* and circ_0026129 in 293A cells.

We then used RT-PCR to validate the expression of circ_0026129, miRNA-15a-5p and *ATP6V1A* in stromal cell exosomes from the three groups of patients. The relative levels of these three RNAs estimated from the RT-PCR analysis are shown in Figure 6A–6C. The validation results were in agreement with the RNA sequencing results.

Finally, we used dual luciferase reporter assays to assess whether miRNA-15a-5p could bind directly to circ_0026129 and *ATP6V1A*. MiRNA-15a-5p mimics reduced the luciferase activities of reporter plasmids containing the potential binding sequences of wild-type circ_0026129 and *ATP6V1A*, but not of mutant circ_0026129 and *ATP6V1A* (Figure 6D, 6E). These results indicated that circ_0026129 may promote *ATP6V1A* expression by competing for miRNA-15a-5p in exosomes. This de-repression may enhance the release of ATP6V1A from endometrial stromal cells to recipient cells in the uterine or abdominal cavity, thus contributing to the pathogenesis of endometriosis.

## DISCUSSION

Exosomes are crucial for both local and systemic cellular communication, due to their ability to transfer RNA between cells [13]. CircRNAs are endogenous noncoding RNAs with widespread distribution and various cellular functions [31, 32]. Recently, circRNAs have been reported to be enriched and stable in exosomes [31, 33], although their involvement in endometriosis has not been demonstrated clearly. Endometriosis is a multifactorial disease, and ectopic implantation depends heavily on other pathological processes, including angiogenesis and immune dysfunction. Thus, exosomal ceRNAs could be important for the communication between endometrial stromal cells and other cells, such as vascular endothelial cells and immune cells.

To explore this possibility, we systematically analyzed exosomal RNA sequencing data, miRNA-target interactions and ceRNA co-expression data in endometriosis for the first time. Based on the ceRNA hypothesis, we constructed an exosomal circRNA-related ceRNA network, and then constructed a hub ceRNA sub-network based on closeness, degree and ceRNA scores. We identified 12 endometriosis-associated exosomal RNA biomarkers, and found that circ_0026129, miRNA-15a-5p and *ATP6V1A* were at the center of the endometriosis-related exosomal ceRNA network. We used PCR to validate the expression of these three RNAs, and employed luciferase reporter assays to verify their molecular binding.

All the ceRNAs (i.e., circRNAs and mRNAs) in a ceRNA network should have three common characteristics [34]: (1) they should be differentially expressed between the comparison groups; (2) they should be the targets of miRNAs; and (3) their interrelationships should adhere to the competition rules of the ceRNA network (for instance, that highly expressed circRNAs sequester miRNAs away from their target mRNAs, thus increasing mRNA expression). Competing RNAs with similar expression exhibit more robust ceRNA effects than ceRNA pairs that are largely differentially expressed [35]. The effectiveness of a ceRNA mainly depends on the number of sponged miRNAs [34], which can be reflected in the ceRNA score and ceRNA P-value.

Presently, there are many methods for ceRNA network construction, most of which only use RNA sequences to predict targets (as in the case of TargetScan) [36]. In our study, we constructed the ceRNA network not only by using bioinformatic tools to predict miRNA targets, but also by considering the competition among the circRNAs, miRNAs and mRNAs and removing interaction pairs with low ceRNA scores. To construct a hub ceRNA network, we identified hub genes with high connectivity to other genes and high ceRNA effects. Circ_0026129, miRNA-15a-5p and *ATP6V1A* were at the center of the network and were differentially expressed between exosomes from endometriosis patients and controls, confirming the reliability of our RNA sequencing results.

Our results were consistent with previous reports. For example, *ATP6V1A* (one of the 12 most important genes identified in our study) has been associated with endometriosis severity and endometrial receptivity [29, 37], and is known to be an oncogene in endometrial cancer [38]. BCL2-like 11, a member of the BCL-2 family, is involved in apoptosis [39], and suppresses invasion in endometriosis by promoting the epithelial-mesenchymal transition [40]. MiRNA-15a-5p downregulates vascular endothelial growth factor A, thus contributing to the pathogenesis of endometriosis [30, 41]. We also identified novel RNAs in endometriosis, such as *AJUBA* and miR-3187-3p. AJUBA is a negative regulator of the Hippo signaling pathway, which promotes cell proliferation and inhibits apoptosis in endometriosis [42, 43]. MiR-3187-3p was previously found to be involved in a ceRNA network that suppresses colorectal cancer cell proliferation [44]. There have been few studies about circRNAs in endometriosis, or about circ_0026129 in general. We found that circ_0026129 could bind to miRNA-15a-5p, which is known to be involved in endometriosis and correlated highly with endometriosis-related mRNAs.

To explore the underlying biological pathways of endometriosis, we performed a GSEA to identify the functions of the DEMs in each of the three comparison sets. Exosomal mRNAs from the EC, EU and Ctrl groups were enriched in different biological processes and pathways, suggesting that exosome secretion uniquely contributes to the etiology of endometriosis. *ATP6V1A* was the core gene in the enriched GO ‘hydrogen ion transmembrane transport’ gene set and the KEGG ‘oxidative phosphorylation’ gene set. HECT and RLD domain containing E3 ubiquitin protein ligase 3 (*HERC3)* was the core gene in the enriched KEGG ‘ubiquitin-mediated proteolysis’ gene set in both the EC vs. Ctrl and EC vs. EU comparisons. Subsequently, we performed GO and KEGG enrichment analyses on the upregulated and downregulated exosomal DEMs that overlapped among the three comparison sets. The upregulated gene *ATP6V1A* was involved in ‘mTOR signaling’, an enriched KEGG pathway known to contribute to the development of endometriosis [45]. Our functional annotation suggested that the identified hub genes were strongly associated with endometriosis, and that the circ_0026129/miRNA-15a-5p/*ATP6V1A* ceRNA network may be important for the development of endometriosis.

To the best of our knowledge, this is the first study to identify a panel of endometriosis-related exosomal RNAs (circRNAs, miRNAs and mRNAs) and their competing relationships based on RNA sequencing data. This biomarker panel may be useful for diagnosing and evaluating the prognosis of endometriosis patients, but further experimental data are needed to delineate the mechanisms of the exosomal ceRNA network and confirm its relevance to the pathogenesis of endometriosis. Our sample size was relatively small. Nevertheless, our study had several strengths. Our RNA sequencing data were subjected to the RNA quality control procedures. Moreover, the DEGs we identified were involved in pathways that have been strongly associated with the development of endometriosis, such as the ‘epithelial to mesenchymal transition’ and the ‘Hippo signaling pathway’ [25, 43]. Additionally, the genes in the top-ranking interaction (circ_0026129/miRNA-15a-5p/*ATP6V1A*) were validated using RT-PCR. Our study has expanded the current understanding of RNA-RNA crosstalk in endometriosis, and our novel method of obtaining exosomal biomarkers has provided potential diagnostic and prognostic tools for this disorder.

## MATERIALS AND METHODS

### Ethics statement

Approval for our study was obtained from the Human Ethics Committee of Second Xiangya Hospital, Central South University. Each participant gave written informed consent in accordance with the Declaration of Helsinki.

### Clinical specimens and cell culture

Primary endometrial stromal cells were obtained from thirteen ovarian endometriomas (EC) and thirteen eutopic endometria (EU) from women with histological diagnoses of stage III-IV endometriosis based on the American Society for Reproductive Medicine classification criteria, as well as from thirteen normal endometria from women who had undergone surgery due to tubal infertility without endometriosis (Ctrl). Three ovarian endometriomas, three eutopic endometria and three normal endometria were used for RNA sequencing, while the other samples were used for RT-PCR analysis. The endometrial tissues were pathologically confirmed in the proliferation stage. All patients were reproductive-age women with regular menstrual cycles, not taking any hormonal contraception or experiencing hormone-dependent disease during the past half year.

After being washed with phosphate-buffered saline, the collected tissues were minced and digested with 0.1% collagenase type I (Sigma-Aldrich, MO, USA) on a rocker for around 1 h (depending on the texture and cell type). Each specimen was centrifuged, and the sediment was cultured in Dulbecco’s modified Eagle’s medium (DMEM) with 10% fetal bovine serum (Thermo Fisher Scientific, MA, USA) in a 5% CO_2_ incubator at 37° C. Immunohistochemical analysis of Vimentin indicated that endometrial stromal cells could be purified to 93-96% at passage 3. The purified endometrial stromal cells were used for exosome isolation.

### Exosome isolation

When the cells were 70-80% confluent, the culture medium was replaced with serum-free DMEM for 48 h. Then, exosomes were extracted from the medium using an ExoQuick-TC exosome isolation kit (SBI, Palo Alto, CA, USA) in accordance with the manufacturer’s protocol. In detail, the serum-free DMEM was collected and centrifuged for cell debris removal. Then, the supernatant was transferred to a sterile vessel, mixed with precipitation solution and incubated at 4° C overnight. On the second day, the mixture was centrifuged, the supernatant was discarded and the exosome pellet was isolated. The exosomes were stored in a -80° C freezer or used immediately for experiments.

### Exosome validation

The exosomes were validated as previously described [46]. The exosomes were visualized using transmission electron microscopy. Paraformaldehyde (4%) was used to fix exosome fractions to carbon-coated electron microscopy nickel grids. Each grid was further fixed with glutaraldehyde, stained with 0.5% aqueous uranyl acetate for 2 h and observed using transmission electron microscopy. Nanoparticle tracking analysis was used to determine the exosome distribution and concentration. Exosomes were diluted in phosphate-buffered saline and loaded into the test cell of a nanoparticle tracking instrument. Then, the nanoparticles were illuminated with a laser, and each sample was tracked automatically by the instrument based on Brownian motion. Additionally, the exosomal markers were analyzed using Western blotting.

### RNA isolation, cDNA library preparation and RNA sequencing

Exosomal RNA sequencing was performed at the Beijing Genomics Institute (BGI, Shenzhen, China). In brief, total RNA was isolated from each exosome sample using Trizol reagent (Life Technologies, Gaithersburg, MD, USA). The quality and quantity of RNA were assessed with an Agilent 2100 Bioanalyzer (Thermo Fisher Scientific), and qRT-PCR was performed. rRNA depletion was performed on the total RNA.

Total RNA from each sample was used to construct a cDNA library. The sequencing libraries were sequenced on an Illumina HiSeq4000 or BGISEQ-500 platform in accordance with the manufacturer’s recommendations. Paired-end reads were generated for miRNAs, whereas single-end reads were generated for circRNAs and mRNAs. Thereafter, low-quality reads and the adaptor sequences of the raw reads were discarded, and clean data were obtained and aligned using Bowtie2. Quality control procedures were used to ensure accurate measurements and correct acquirements. The mapped reads were further assembled using Cufflinks. DEMs, DEMis and DECs with statistical significance were identified using the DESeq package with the threshold of a fold-change > 2 and a corrected P-value < 0.001.

### GO and KEGG pathway enrichment analyses of DEGs

In order to annotate the functions of the DEGs, we performed GO and KEGG enrichment analyses using ‘phyper’ [47], a hypergeometric distribution function in R. P-values < 0.05 were considered statistically significant.

### GSEA

GSEA (http://www.broadinstitute.org/gsea/index.jsp) was performed on the EC, EU and Ctrl groups to investigate the biological functions and pathways involved in endometriosis. Java GSEA implementation was used for the analysis. The C2 (curated KEGG) and C5 (GO biological process) reference gene sets were obtained from the Molecular Signatures Database. Significant gene sets with P-values < 0.05 and false discovery rates < 25% were identified.

### Prediction and refinement of miRNA-target interaction pairs

We first predicted miRNA-target interaction pairs using bioinformatic databases. MiRanda (http://miranda.org.uk), TargetScan (http://www.targetscan.org) and RNAhybrid-2.1.2 were used to predict miRNA-mRNA interactions. The StarBase database [48] was used to predict miRNA-circRNA interactions. MiRNA-mRNA and miRNA-circRNA pairs that overlapped with DEGs in the three comparison sets were selected.

We then predicted miRNA-target interaction pairs based on correlation coefficients. We evaluated the Pearson correlation coefficients between miRNA levels and circRNA/mRNA levels using the ‘cor’ function in R. Negative correlations between miRNAs and their targets (R < -0.4) were selected [34].

Finally, to enhance the reliability of prediction, we selected the overlapping miRNA–target interaction pairs between the bioinformatic analysis and the correlation coefficient analysis. Pairs that shared one miRNA were considered as candidate circRNA-miRNA-mRNA interactions for the subsequent ceRNA prediction.

### Prediction and refinement of ceRNA pairs

We first predicted ceRNA pairs by evaluating the Pearson correlation coefficients between circRNA and mRNA levels. Positive correlations between circRNAs and mRNAs (R > 0.4) were selected. We chose ceRNA (circRNA-mRNA) pairs based on these co-expression values and circRNA-miRNA-mRNA competing interactions.

We then predicted ceRNA pairs by computing ceRNA scores [35]. The ceRNA score was calculated as the number of shared miRNAs between a ceRNA pair (circRNA-mRNA) divided by the total number of miRNAs targeting the individual candidate genes [35]. The P-value for each potential ceRNA pair was determined using a hypergeometric test based on the previously described formula $P=\sum_{i=c}^{\min\;(K,n)}\left(\begin{array}{c} K\\ i \end{array}\right)\left(\begin{array}{c} N-K\\ n-i \end{array}\right)/\left(\begin{array}{c} N\\ n \end{array}\right),$ where K is the number of miRNAs interacting with a specified gene, n is the number of miRNAs interacting with the ceRNA of the specified gene, c is the number of shared miRNAs between these two genes, and N is the total number of miRNAs [28]. CeRNA pairs with corrected P-values > 0.05 or ceRNA scores ≤ 0.3 were removed.

Finally, we refined the ceRNA pairs based on both their Pearson correlation coefficients and their ceRNA scores.

### Construction of co-expression and ceRNA networks

CeRNA pairs were used to construct a circRNA-mRNA co-expression network. Then, a circRNA-miRNA-mRNA ceRNA network was constructed based on the ceRNA pairs that shared miRNAs. Genes with high degrees of connectivity tended to be at the center of the ceRNA network, so we calculated the degrees of connectivity for nodes in the ceRNA network. Cytoscape was used to visualize the ceRNA network. Larger nodes were used to represent genes with higher degrees of connectivity.

### Topological analysis and construction of the hub ceRNA sub-network

A topological analysis of circRNAs in the ceRNA network was conducted to identify the hub nodes of endometriosis. Topological parameters (the ceRNA score, ceRNA P-value, degree, and closeness) were calculated using the Cytoscape plug-in CentiScaPe. The top-ranking circRNAs for these four topological parameters were compared, and those that overlapped were used to construct a hub ceRNA sub-network. A Venn diagram was used to identify genes that overlapped between the hub ceRNA sub-network and the list of genes with higher degrees of connectivity. Cytoscape was used to visualize the sub-network. Larger nodes were used to represent overlapping hub genes.

### Reverse transcription and polymerase chain reaction (RT-PCR) analysis

To validate the RNA sequencing data, we performed RT-PCR on stromal cell exosomes from the EC (n=10), EU (n=10), and Ctrl (n=10) groups. A miRNA/HiFiScript cDNA Synthesis Kit (CWBio Co. Ltd., Beijing, China) was used to reverse-transcribe total RNA into first-strand cDNA. Ultra SYBR Mixture (Low ROX) (CWBio Co. Ltd.) was used to amplify the cDNA samples. The primers are listed in Supplementary Table 4. U6 was used as an internal control for miRNAs, while *β-actin* was used as an internal control for circRNAs and mRNAs [49]. The primers were designed using primer5. Candidate circRNAs were characterized by RT-PCR using the outward-facing primers annealing at the distal ends of the circRNAs [50, 51]. The sequence specificity was verified through BLAST searches*.* The mean Ct value of all the technical replicates for each sample was assessed, and relative RNA expression was calculated using the 2^-ΔΔCt^ method.

### Luciferase reporter assay

Reporter plasmids expressing wild-type (WT) and mutant (MUT) sequences for the 3′ untranslated regions (3′UTRs) of circ_0026129 (pHG-MirTarget-circ_0026129-3′UTR-WT and pHG-MirTarget-circ_0026129-3′UTR-MUT, respectively) and *ATP6V1A* (pHG-MirTarget-ATP6V1A-3′UTR-WT and pHG-MirTarget-ATP6V1A-3′UTR-MUT, respectively) were obtained from HonorGene Biotechnology, along with miRNA-15a-5p mimics and negative control (miRNA-15a-5p-NC) plasmids. To confirm that miRNA-15a-5p could bind directly to the 3′UTRs of circ_0026129 and *ATP6V1A*, we co-transfected 293A cells with the pHG-MirTarget-circ_0026129-3′UTR-WT, pHG-MirTarget-circ_0026129-3′UTR-MUT, pHG-MirTarget-ATP6V1A-3′UTR-WT or pHG-MirTarget-ATP6V1A-3′UTR-MUT reporter plasmids and either miRNA-15a-5p mimics or miRNA-15a-5p-NC. Twenty-four hours after transfection, luciferase activity was measured on a Dual-Luciferase Reporter Assay System (Promega, Madison, WI, USA). The ratio of luciferase activity was calculated for each sample, in accordance with the manufacturer’s instructions.

### Statistical analysis

The data presented in our study are representative of three independent experiments. The results were analyzed using one-way analysis of variance with Fisher’s least significant difference post hoc test in SPSS version 23 (SPSS Inc., Chicago, IL, USA). Data are presented as the mean ± standard deviation. P-values < 0.05 were considered statistically significant.

### Data availability

The data that support the findings of this study are available from the corresponding author upon reasonable request.

## Supplementary Material

Supplementary Figures

Supplementary Tables
