# Supplementary material for: Construction and topological analysis of an endometriosis-related exosomal circRNA-miRNA-mRNA regulatory network
Source: Aging (Albany NY). 2021 Apr 26;13(9):12607–30. doi: 10.18632/aging.202937 (PMC8148458; doi:10.18632/aging.202937)
Supplement: Supplementary Figures [file aging-13-202937-s001.pdf]

SUPPLEMENTARY FIGURES

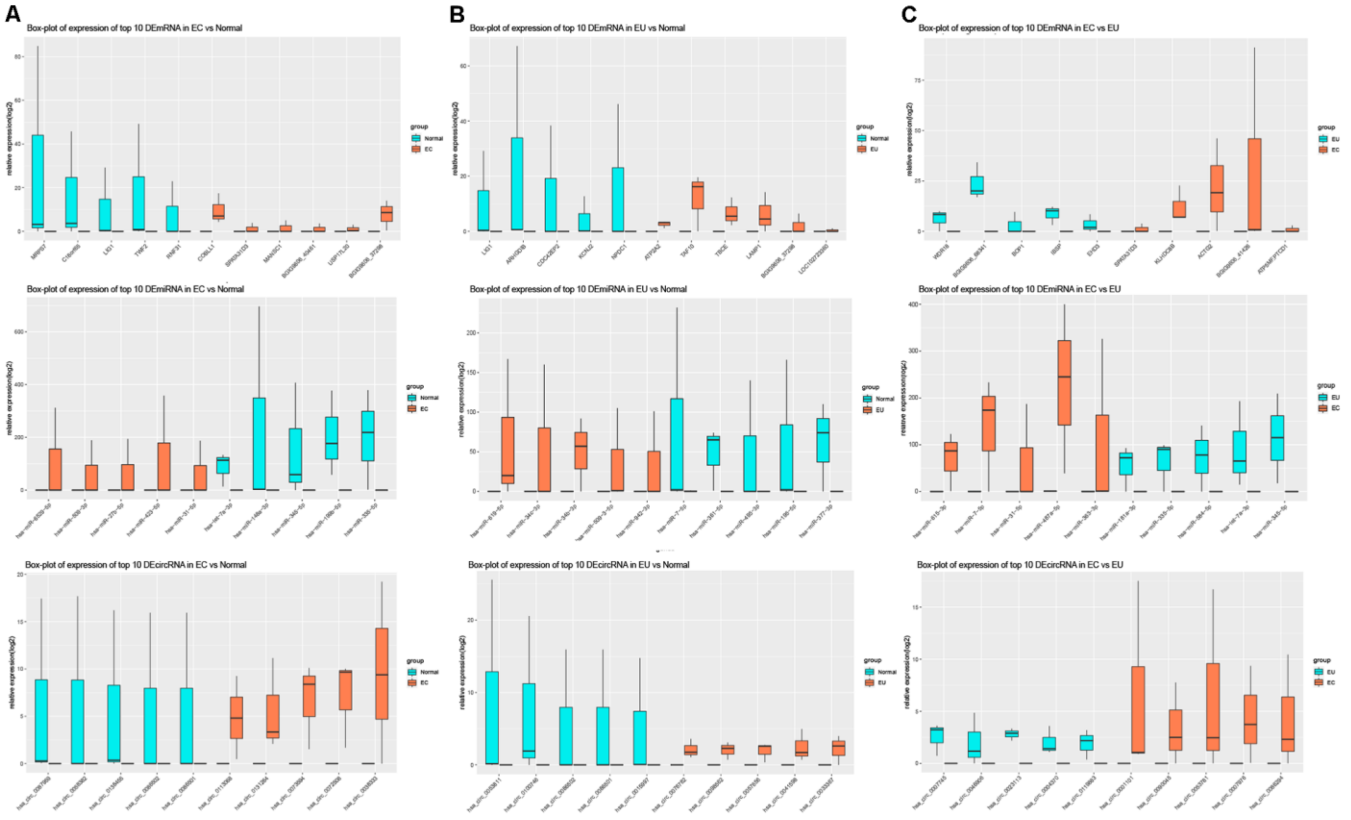

Supplementary Figure 1. Histogram showing the expression of the top 10 DECs, DEMis and DEMs between the EC and Ctrl (**A**), EU and Ctrl (**B**) and EC and EU groups (**C**).

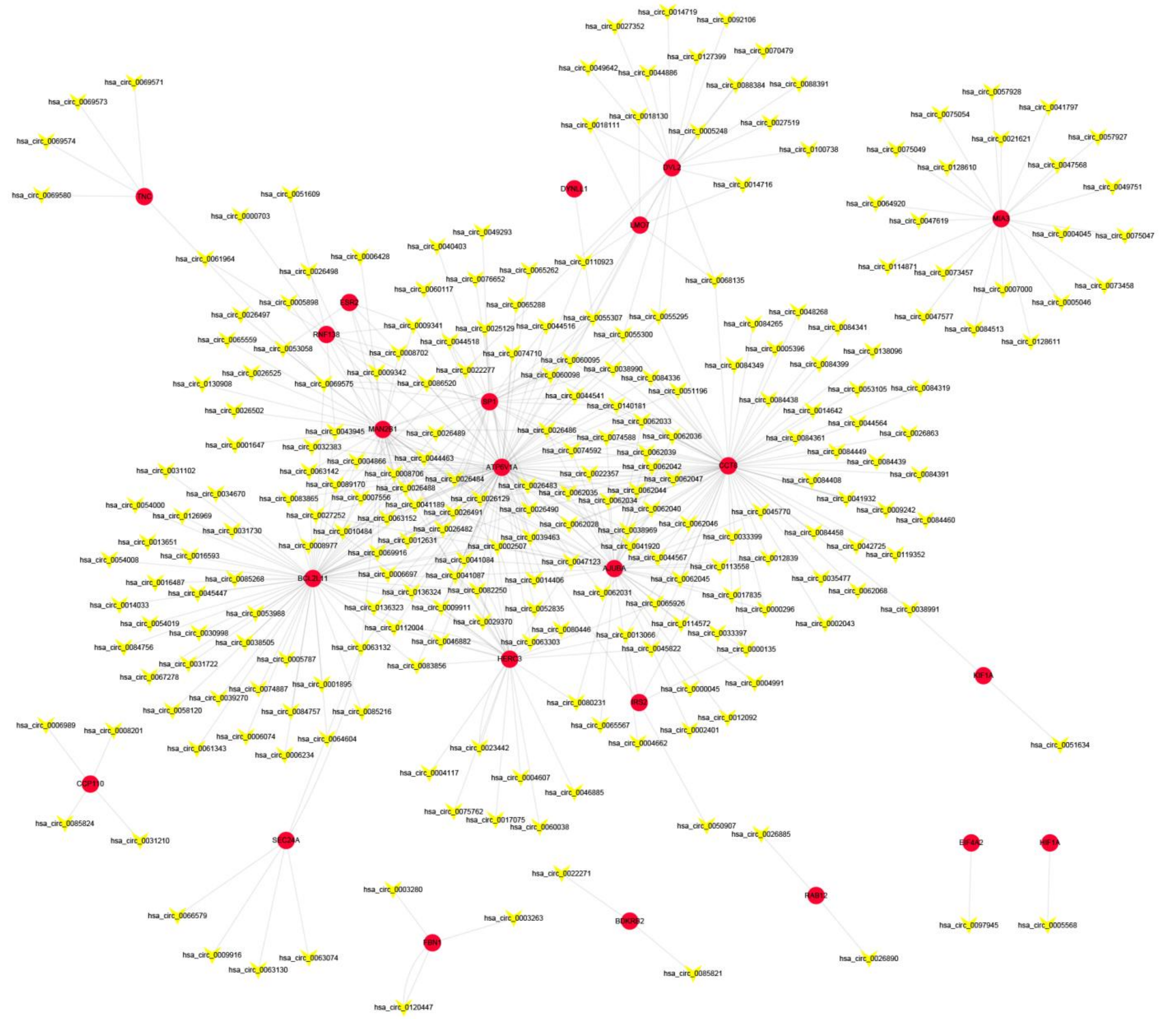

Supplementary Figure 2. The circRNA-mRNA co-expression network. Yellow V-shaped nodes represent DECs, and red circular nodes represent DEMs.
