# Supplementary material for: Construction and topological analysis of an endometriosis-related exosomal circRNA-miRNA-mRNA regulatory network
Source: Aging (Albany NY). 2021 Apr 26;13(9):12607–30. doi: 10.18632/aging.202937 (PMC8148458; doi:10.18632/aging.202937)
Supplement: Supplementary Tables [file aging-13-202937-s002.pdf]

SUPPLEMENTARY TABLES

Supplementary Table 1. GSEA of the DEMs in three comparison sets.

GROUP	NAME	NG*	NES	NOM p-val	FDR q-val
EC vs. Ctrl	**GO_POST_GOLGI_VESICLE_MEDIATED_TRANSPORT**	**79**	**2.13**	**0**	**0.0338**
	GO_REGULATION_OF_CARDIAC_CONDUCTION	53	2.103	0	0.0266
	GO_REGULATION_OF_P38MAPK_CASCADE	22	2.091	0	0.0205
	GO_NEGATIVE_REGULATION_OF_STRESS_ACTIVATED_PROTEIN_KINASE_SIGNALING_CASCADE	39	2.049	0	0.0356
	GO_MODULATION_BY_SYMBIONT_OF_HOST_CELLULAR_PROCESS	26	2.04	0.001	0.0344
	KEGG_VASCULAR_SMOOTH_MUSCLE_CONTRACTION	99	1.791	0	0.2194
	**KEGG_UBIQUITIN_MEDIATED_PROTEOLYSIS**	**132**	**1.77**	**0**	**0.1554**
	KEGG_PROTEASOME	42	1.764	0.001	0.1068
	KEGG_ALZHEIMERS_DISEASE	144	1.747	0	0.0939
	KEGG_CYSTEINE_AND_METHIONINE_METABOLISM	32	1.732	0.01	0.0907
EU vs. Ctrl	GO_POSITIVE_REGULATION_OF_DNA_TEMPLATED_TRANSCRIPTION_INITIATION	21	2.156	0	0.0362
	GO_REGULATION_OF_TRANSCRIPTION_INITIATION_FROM_RNA_POLYMERASE_II_PROMOTER	20	2.111	0	0.042
	**GO_HYDROGEN_ION_TRANSMEMBRANE_TRANSPORT**	**89**	**2.1**	**0**	**0.0345**
	GO_REGULATION_OF_DNA_TEMPLATED_TRANSCRIPTION_INITIATION	28	2.079	0	0.035
	GO_SNRNA_METABOLIC_PROCESS	78	2	0	0.0956
	**KEGG_OXIDATIVE_PHOSPHORYLATION**	**107**	**2.25**	**0**	**0**
	KEGG_BASAL_TRANSCRIPTION_FACTORS	33	1.92	0	0.0385
	KEGG_HUNTINGTONS_DISEASE	160	1.886	0	0.0343
	KEGG_PROTEASOME	41	1.871	0	0.0319
	KEGG_PARKINSONS_DISEASE	107	1.855	0	0.032
EC vs. EU	GO_CEREBELLAR_CORTEX_MORPHOGENESIS	28	2.229	0	0.0176
	**GO_ADHERENS_JUNCTION_ORGANIZATION**	**64**	**2.1**	**0**	**0.0743**
	GO_POSITIVE_REGULATION_OF_VIRAL_GENOME_REPLICATION	30	2.08	0	0.0624
	GO_MACROPHAGE_ACTIVATION	24	2.062	0.001	0.0559
	GO_RESPONSE_TO_DIETARY_EXCESS	16	2.055	0	0.0484
	KEGG_VASCULAR_SMOOTH_MUSCLE_CONTRACTION	115	1.791	0	0.2194
	**KEGG_UBIQUITIN_MEDIATED_PROTEOLYSIS**	**135**	**1.77**	**0**	**0.1554**
	KEGG_PROTEASOME	44	1.764	0.001	0.1068
	KEGG_ALZHEIMERS_DISEASE	157	1.747	0	0.0939
	KEGG_CYSTEINE_AND_METHIONINE_METABOLISM	34	1.732	0.01	0.0907

Note: The 6 most common gene sets were highlighted in table. GSEA: Gene Set Enrichment Analysis. DEMs: Differentially expressed mRNAs. Three comparison sets: EC vs. EU, EU vs. Ctrl and EC vs. EU. Ctrl: exosomes secreted by stromal cells of normal endometria from patient without endometriosis. EC: exosomes secreted by stromal cells of ovarian endometriomas from patient with endometriosis. EU: exosomes secreted by stromal cells of eutopic endometria from patient with endometriosis.

Supplementary Table 2. The top enriched GO BP terms and KEGG pathway terms of upregulated DEMs.

GO term	GO term description	GO term level 1	GO term level 2	Term gene num	Total gene num	P value
GO:0001837	epithelial to mesenchymal transition	biological_process	developmental process	51	24363	9E-05
GO:0010950	positive regulation of endopeptidase activity	biological_process	regulation of biological process	16	24363	3E-04
GO:2000562	negative regulation of CD4-positive, alpha-beta T cell proliferation	biological_process	biological adhesion	7	24363	3E-04
GO:0043687	post-translational protein modification	biological_process	metabolic process	438	24363	4E-04
GO:1902443	negative regulation of ripoptosome assembly involved in necroptotic process	biological_process	regulation of biological process	2	24363	4E-04
GO:0002062	chondrocyte differentiation	biological_process	developmental process	53	24363	8E-04
GO:0030177	positive regulation of Wnt signaling pathway	biological_process	regulation of biological process	53	24363	8E-04
GO:0010718	positive regulation of epithelial to mesenchymal transition	biological_process	developmental process	56	24363	0.001
GO:0032922	circadian regulation of gene expression	biological_process	metabolic process	78	24363	0.001
GO:0032774	RNA biosynthetic process	biological_process	metabolic process	3	24363	0.001
GO:1905426	positive regulation of Wnt-mediated midbrain dopaminergic neuron differentiation	biological_process	developmental process	3	24363	0.001
GO:0042698	ovulation cycle	biological_process	reproduction	11	24363	0.001
GO:1990253	cellular response to leucine starvation	biological_process	response to stimulus	11	24363	0.001
GO:0008286	insulin receptor signaling pathway	biological_process	regulation of biological process	104	24363	0.002
GO:0043123	positive regulation of I-kappaB kinase/NF-kappaB signaling	biological_process	regulation of biological process	239	24363	0.002
GO:0140052	cellular response to oxidised low-density lipoprotein particle stimulus	biological_process	response to stimulus	12	24363	0.002
GO:0038061	NIK/NF-kappaB signaling	biological_process	regulation of biological process	26	24363	0.002
GO:0043488	regulation of mRNA stability	biological_process	metabolic process	109	24363	0.002
GO:0006468	protein phosphorylation	biological_process	metabolic process	850	24363	0.002
GO:1903955	positive regulation of protein targeting to mitochondrion	biological_process	regulation of biological process	45	24363	0.002
**KEGG pathway term**	**KEGG pathway term description**	**KEGG pathway term level 1**	**KEGG pathway term level 2**	**Term gene num**	**Total gene num**	**P value**
410	beta-Alanine metabolism	Metabolism	Metabolism of other amino acids	42	15870	0.001
1100	Metabolic pathways	Metabolism	Global and overview maps	1923	15870	0.001
71	Fatty acid degradation	Metabolism	Lipid metabolism	57	15870	0.002
280	Valine, leucine and isoleucine degradation	Metabolism	Amino acid metabolism	64	15870	0.003
1130	Biosynthesis of antibiotics	Metabolism	Global and overview maps	327	15870	0.004
900	Terpenoid backbone biosynthesis	Metabolism	Metabolism of terpenoids and polyketides	34	15870	0.011
4013	MAPK signaling pathway	Environmental Information Processing	Signal transduction	103	15870	0.013
940	Phenylpropanoid biosynthesis	Metabolism	Biosynthesis of other secondary metabolites	3	15870	0.014
4072	Phospholipase D signaling pathway	Environmental Information Processing	Signal transduction	197	15870	0.014
4910	Insulin signaling pathway	Organismal Systems	Endocrine system	197	15870	0.014
4912	GnRH signaling pathway	Organismal Systems	Endocrine system	118	15870	0.018
4022	cGMP-PKG signaling pathway	Environmental Information	Signal transduction	228	15870	0.023

		Processing				
4062	Chemokine signaling pathway	Organismal Systems	Immune system	252	15870	0.031
5110	Vibrio cholerae infection	Human Diseases	Infectious diseases: Bacterial	62	15870	0.035
1212	Fatty acid metabolism	Metabolism	Global and overview maps	69	15870	0.042
4926	Relaxin signaling pathway	Organismal Systems	Endocrine system	164	15870	0.043
1110	Biosynthesis of secondary metabolites	Metabolism	Global and overview maps	558	15870	0.048
4152	AMPK signaling pathway	Environmental Information Processing	Signal transduction	175	15870	0.05

Note: GO BP: Gene ontology analysis of biological process. KEGG: Kyoto Encyclopedia of Genes. DEM: differentially expressed mRNA among the three comparison sets (EC vs. EU, EU vs. Ctrl, EC vs. EU).

Supplementary Table 3. The top enriched GO BP terms and KEGG pathway terms of downregulated DEMs.

GO term	GO term description	GO term level 1	GO term level 2	Term gene num	Total gene num	P value
GO:0006210	thymine catabolic process	biological_process	metabolic process	4	24363	1E-04
GO:0061179	negative regulation of insulin secretion involved in cellular response to glucose stimulus	biological_process	regulation of biological process	10	24363	0.001
GO:0001819	positive regulation of cytokine production	biological_process	regulation of biological process	41	24363	0.001
GO:0048643	positive regulation of skeletal muscle tissue development	biological_process	developmental process	15	24363	0.003
GO:0042445	hormone metabolic process	biological_process	metabolic process	18	24363	0.004
GO:0046475	glycerophospholipid catabolic process	biological_process	metabolic process	19	24363	0.004
GO:0035518	histone H2A monoubiquitination	biological_process	metabolic process	20	24363	0.005
GO:0090557	establishment of endothelial intestinal barrier	biological_process	developmental process	21	24363	0.005
GO:0000415	negative regulation of histone H3-K36 methylation	biological_process	metabolic process	1	24363	0.005
GO:0002731	negative regulation of dendritic cell cytokine production	biological_process	regulation of biological process	1	24363	0.005
GO:0006743	ubiquinone metabolic process	biological_process	metabolic process	1	24363	0.005
GO:0019859	thymine metabolic process	biological_process	metabolic process	1	24363	0.005
GO:0034143	regulation of toll-like receptor 4 signaling pathway	biological_process	immune system process	1	24363	0.005
GO:0034213	quinolinate catabolic process	biological_process	metabolic process	1	24363	0.005
GO:0036138	peptidyl-histidine hydroxylation	biological_process	metabolic process	1	24363	0.005
GO:0042265	peptidyl-asparagine hydroxylation	biological_process	metabolic process	1	24363	0.005
GO:0042790	nucleolar large rRNA transcription by RNA polymerase I	biological_process	metabolic process	1	24363	0.005
GO:0045221	negative regulation of FasL biosynthetic process	biological_process	metabolic process	1	24363	0.005
GO:0071166	ribonucleoprotein complex localization	biological_process	localization	1	24363	0.005
GO:0071881	adenylate cyclase-inhibiting adrenergic receptor signaling pathway	biological_process	signaling	1	24363	0.005
**KEGG pathway term**	**KEGG pathway term description**	**KEGG pathway term level 1**	**KEGG pathway term level 2**	**Term gene num**	**Total gene num**	**P value**
410	beta-Alanine metabolism	Metabolism	Metabolism of other amino acids	42	15870	0.001
1100	Metabolic pathways	Metabolism	Global and overview maps	1923	15870	0.001
71	Fatty acid degradation	Metabolism	Lipid metabolism	57	15870	0.002

280	Valine, leucine and isoleucine degradation	Metabolism	Amino acid metabolism	64	15870	0.003
1130	Biosynthesis of antibiotics	Metabolism	Global and overview maps	327	15870	0.004
900	Terpenoid backbone biosynthesis	Metabolism	Metabolism of terpenoids and polyketides	34	15870	0.011
4013	MAPK signaling pathway - fly	Environmental Information Processing	Signal transduction	103	15870	0.013
940	Phenylpropanoid biosynthesis	Metabolism	Biosynthesis of other secondary metabolites	3	15870	0.014
4072	Phospholipase D signaling pathway	Environmental Information Processing	Signal transduction	197	15870	0.014
4910	Insulin signaling pathway	Organismal Systems	Endocrine system	197	15870	0.014
4912	GnRH signaling pathway	Organismal Systems	Endocrine system	118	15870	0.018
4022	cGMP-PKG signaling pathway	Environmental Information Processing	Signal transduction	228	15870	0.023
4062	Chemokine signaling pathway	Organismal Systems	Immune system	252	15870	0.031
5110	Vibrio cholerae infection	Human Diseases	Infectious diseases: Bacterial	62	15870	0.035
1212	Fatty acid metabolism	Metabolism	Global and overview maps	69	15870	0.042
4926	Relaxin signaling pathway	Organismal Systems	Endocrine system	164	15870	0.043
1110	Biosynthesis of secondary metabolites	Metabolism	Global and overview maps	558	15870	0.048
4152	AMPK signaling pathway	Environmental Information Processing	Signal transduction	175	15870	0.05

Supplementary Table 4. Primers used in this study.

Names	Sequences(5'-3')
hsa-miR-15a-5p	Forward: GGGTCGTAGCAGCACATAATGG
hsa-circ_0026129	Forward: AAGTATTCCCTGTCTTAAGCCGAC
	Reverse: CTTGCCATAATCAACTGAGAGACG
ATP6V1A	Forward: ACAGCCTCTGGGTCCTCGGTCG
	Reverse: CCTGAGACCCCATGCACATAACCAA
U6	Forward: CTCGCTTCGGCAGCACA
	Reverse: AACGCTTCACGAATTTGCGT
GAPDH	Forward: ACAGCCTCAAGATCATCAGC
	Reverse: GGTCATGAGTCCTTCCACGAT
